# When the regime goes local: Data on case description, summary of cases, and Swiss institutional soil regime

**DOI:** 10.1016/j.dib.2019.104341

**Published:** 2019-09-16

**Authors:** François-Xavier Viallon

**Affiliations:** Swiss Graduate School of Public Administration, University of Lausanne, 1015, Lausanne, Switzerland

**Keywords:** Land use policy, Policy implementation, Qualitative comparative analysis, Institutional resource regime, Local regulatory arrangement, Property rights, Policy expertise

## Abstract

The article presents the data related to the study “When the regime goes local: Local regulatory arrangements and land use sustainability” (Viallon et al., 2019). The data collection process comprises legal and document analysis as well as 35 qualitative interviews. Data analysis includes: 1) reconstructing the Swiss institutional soil regime, 2) tracing of land use planning and land use changes over the past decades in the areas selected for analysis, 3) identifying contextual factors influencing the local regulatory arrangements, 4) presenting the constraints of superior law applying to each case, 5) analysing actors’ games, the strategies they pursue, and the resources and instruments they mobilize, 6) describing and qualifying the local regulatory arrangement between actors. Data can be reused for future analyses of land use policy processes and their comparison in Switzerland and abroad.

**Table:** Specifications Table

Subject	Sociology and Political Science
Specific subject area	Land use policy implementation
Type of data	Table Text
How data were acquired	Document analysis, semi-structured interviews, press review, case law review, keyword analysis in national and regional newspaper databases.
Data format	Raw Synthesized Coded
Parameters for data collection	1. Important changes in land use planning and land use over the past 25 years; 2. Presence of oversized building zones; 3. Different property rights settings (e.g. private, public, readjustment of land plots); 4. Important issues around ecological and economic added and/or reduced value; 5. Presence of conflicts; 6. Use of land use different policy instruments.
Description of data collection	The first step of data collection was to conduct a legal review of private and public laws applying to soil in Switzerland. Reviewed legislation included property rights (civil code), land use planning acts, and soil protection acts. Second, we conducted a literature review, a press review, a case law review, and a keyword analysis in national and regional newspaper databases to identify policy problems and cases. The third step consisted in collecting case related documents in the cantonal and communal administration's planning offices and conducting interviews with the identified stakeholders for each case analyzed.
Data source location	City: Berne Country: Switzerland 46.94847, 7.45635 City: Wiedlisbach Country: Switzerland 47.25208, 7.64634 City: Huttwil Country: Switzerland 47.11324, 7.85039 City: Niederbipp Country: Switzerland 47.26692, 7.69581 City: Lausanne Country: Switzerland 46.5267096, 6.6025582 City: Cheseaux Country: Switzerland 46.5854324, 6.6061393
Data accessibility	Repository name: FORSbase Data identification number: 1001 Direct URL to data: https://forsbase.unil.ch/datasets/dataset-public-detail/15288/1799/ Repository name: SERVAL Data identification number: BIB_E738FB9AFB2A Direct URL to data: https://serval.unil.ch/resource/serval:BIB_E738FB9AFB2A.P001/REF
Related research article	François-Xavier Viallon, Rémi Schweizer, Frédéric Varone When the regime goes local: Local regulatory arrangements and land use sustainability Environmental Science & Policy 10.1016/j.envsci.2019.02.010

**Table:** 

**Value of the Data** • Provides a means to operationalize the Institutional Resource Regime conceptual framework and local regulatory arrangements • Other researchers working in the field of policy implementation or conducting qualitative comparative analysis can benefit from the data, as they can reuse the methodology and coding used • The data grants additional in depth qualitative insights on the cases and provides transparency over the coding process • The data can benefit future research on (land use) policy implementation for comparison

## 1 Data

The shared data is subdivided into two tables. Table 1 describes the policy problem that applies to each case. It provides preliminary information to the reader required to understand the commented data in Table 2. Table 2 describes the coding of the variables for each case analyzed:

• Local Regulatory Arrangement (LRA) is the dependent variable. It is coded ‘0’ for cases of diversion and circumvention, ‘1’ for cases of implementation and innovation;
• ‘Previous use of instrument (PI)’ is coded ‘0’ in the absence of previous use of the instrument, or when implementation is delegated to communes, and ‘1’ when the instrument is used within the canton or its implementation is mandatory for communes;
• ‘Already existing development rights within the case's area (DR)’ is coded ‘0’ if negotiation concerns already existing development rights, and ‘1’ if new or additional development rights are negotiated;
• ‘Available resources of authority (RA)’ is coded ‘0’ when no information and personnel is available or resources is used, and ‘1’ when information and personnel are available and used;
• ‘Resources of target group (RT)’ is coded 1 when ‘no information and personnel is available or the resource is not used’ and ‘0’ when ‘information and personnel is available and used’;
• ‘Fragmentation of ownership within the case area (FO)’ is coded ‘0’ in case multiple owners (10 or more) are involved in the policy process, ‘1’ in case few owners (5 or less) are involved.

**Table 1:** List of cases and their related policy problem.

Case	Policy problem
1 - Land hoarding	Lack of building zone available for development and hoarded by landowners
2 - Joint development	Lack of building zone available for development, rural exodus
3 - Building zone reduction	Lack of building zone available for development and hoarded by landowners
4 - Landfill development	Water infiltration in polluted soil causing environmental damage
5 - Value recovery A	Costs of communal infrastructure necessary for development
6 - Polluted soil development	Contaminated soils causing environmental damage and generating high development costs
7 - Major railways accident	Security risks induced by proximity of land to railways
8 - Value recovery B	Costs of communal infrastructure necessary for development
9 - Energy provision	High level of energy consumption for the heating and use of constructions
10 - Public infrastructure provision	Lack of available plots in central location for public infrastructure
11 - Property relocation	Lack of available plots in central location for public infrastructure
12 - Coordination of property and zoning	Lack of coordination between plot boundaries and zoning ordinance

**Table 2:** Commented table of the coded data used for qualitative comparative analysis in Viallon, Schweizer, Varone [1].

Case	LRA	Previous use of instrument (PI)	Existing development rights (DR)	Resources of authority (RA)	Resources of target group (RT)	Fragmen-tation of ownership (FO)
1	1 = Contractual agrements on land service and development	0 = Commune defined new rules in local building regulations	1 = No development right granted prior to negotiation	1 = Use of resources to negotiate with landowners	1 = No use of resources	1 = Two landowners
2	1 = Land service and development	1 = Commune and landowner used zoning and leasehold together in the past	1 = No development right granted prior to negotiation	1 = Use of shared resources to elaborate leasehold contract	0 = Use of shared resources	1 = One semi-public landowner
3	0 = Building zone reduction and no land development	1 = Building zone reductions in the past	0 = Land zoned constructible in the 1970s	0 = No use of information regarding compensation payment	0 = Use of information to threaten the commune	1 = Three landowners
4	1 = Land development and reduction the of the exfiltration of pollutants	1 = Canton implemented contaminated sites ordinance before	0 = Development rights granted prior to development	1 = Use of information to measure groundwater pollution changes	0 = Use of shared information	1 = One developer
5	1 = Value recovery	0 = First time introduction specific to the extension of the industrial zone	1 = No development right granted prior to negotiation	1 = Use of resources to transpose instrument in local regulations	1 = No use of resources	0 = Twelve landowners
6	1 = Relocation of development rights and reduction of excavations	1 = Previous implementation of contaminated sites ordinance	1 = Limited development rights granted prior to negotiation (industrial zone)	1 = Use of resources with landowners to relocate development rights where soil is less polluted	0 = Use of resources shared with commune	1 = Two landowners and two infrastructure owners
7	0 = Gain of additional development rights	0 = Absence of methodology for risk assessment	0 = Use restrictions imposed after negotiations	1 = Use of resources to elaborate MAO restriction criteria	0 = Use of resources to provide risk assessment report	1 = Two landowners
8	0 = Reduction of financial amount to be paid	0 = First time use of extended land service tax	0 = Development rights granted prior to instrument implementation	0 = No use of resources to check costs calculation provided by landowners	0 = Use of resources to inflate developennt costs	1 = Two landowners
9	0 = Circumvention of energy targets	0 = First time use of energy plans in a redevelopment project	0 = District heating extended and planned before planning works began	0 = No information on future share of renewable energy of district heating	1 = No information on future share of renewable energy of district heating	1 = Two landowners and three infrastructure owners
10	1 = Development of museum close to transport infrastructure	1 = Previous exchanges of land plots	1 = Granting of development rights as condition of the deal	1 = Use of resources to negotiate land plot values	0 = Use of resources to negotiate land plot values	1 = Two landowners
11	1 = Developmnt of school close to transport infrastructure	0 = No previous exchange of plots in a betterment procedure	1 = No development right granted prior to negotiation	1 = Use of resources to anticipate future needs	1 = No use of resrouces	0 = Fourteen landowners
12	1 = Coordination between zoning and plot boundaries	0 = No use of land improvement syndicate before	1 = No development right granted prior to negotiation	1 = Use of resources to anticipate future needs	1 = No use of resrouces	1 = Five landowners and commune

To gain a full understanding of the coding, we suggest interested readers to consult the data set [2] attached to the present article. The data set provides:

• A detailed description of the twelve cases analyzed in the main research article [1]. For each case, we describe the local policy problem faced by authorities, the actors involved in the land use policy implementation process, the strategies they adopted, and the local regulatory arrangement they negotiated;
• A summary of the twelve land use policy cases analyzed;
• A synthesis of the evolution of the Swiss institutional soil regime.

The synthesis of the Swiss institutional soil regime described in the data set [2] rests upon the institutional resource regime framework [3] used in the research article [1]. The institutional resource regime framework distinguishes among the property rights system and the policy design. The main modifications of the Swiss soil property rights system shown in the data set are based on the work of Nahrath [4]. Using a bundle of rights approach [5], [6], the author identifies modifications of the soil property rights system and distinguishes their impact on use, disposal and formal possession rights. Further, the data set shows the evolution of the policy design of the resource soil according to Nahrath [4] and Viallon [7]. Changes to the policy design include evolution of the policy problem, and of the related causal and intervention hypotheses [8].

## 2 Experimental design, materials, and methods

In this section, we expose the criteria used for area and case selection, we list the type of secondary data collected (Table 3), and name the functions of the representatives we interviewed (Table 4). The empirical material and methodology exposed here are those of the author's PhD research [7]. Both the research article [1] and the PhD use the Institutional resource regime framework [3] as a theoretical approach.

**Table 3:** Type of secondary data collected.

Preliminary exam documents and/or planning documents	These documents were submitted by the communes to the canton prior to the revision of their building regulations: • (Preliminary) Reports on past and future communal development; • New building regulations as proposed by the commune (structure plans, construction prescriptions, zoning plans, landscape and nature protection plans, land service plans); • Reports on the new building regulations justifying how the context changes and the revised regulations tackle these changes; • Protocols and presentations of the communal executive body and legislative body modifying and approving the new documents; • Opposition raised by landowners, organisations, and other interested parties, and protocols of negotiations (if available); • Reports of the cantonal planning office and other offices involved in the approval procedure; • Written correspondence between the canton, commune, urban planner, and other actors and notes relating to given phone calls; • Report on public consultation procedures;
Approval documents	E.g. approval decisions or decrees by the cantonal authority, final building regulations;
Oppositions and appeals	Potential opposition and appeals against communal and cantonal decisions;
Grey literature	E.g. legislation, reports and statistical information published by communal, cantonal services, federal offices (spatial planning offices, water protection office, finance department, audit reports), contracted planning companies, soil experts, environmental impact assessments, statistics

**Table 4:** Types of representatives interviewed.

Planners	The planner contracted by the commune(s) for establishing the local development plans and revising the building regulations
Local political-administrative representative(s)	The chief of the communal executive body and/or the communal administrator in charge of planning and construction, general administration of the commune
Target groups	Landowners, developers, owners of infrastructure, companies targeted by regulation
Supervisory authorities	The cantonal planner in charge of approving communal land use planning documents, and her/his co-workers (legal officer, planner from the section dedicated to cantonal or regional land use planning); other cantonal offices involved (office for water protection, economic promotion, energy)
Third parties	E.g. environmental organisations, landscape protection associations, journalists

The selection, collection, and sorting of data relies on two levels of analysis. The first level of analysis is the regional area, i.e. a functional perimeter as defined by the Federal statistical office [15]. It provides information on the type of location within which data collection occurs, and the policy problems faced in these areas. The second level of analysis is the local level of land use policy implementation, i.e. where the cases analyzed have occurred. For planning authorities, a large part of land use policy implementation occurs on communal level. Depending on the policies involved (e.g., soil and water protection, coordination of urbanization and transport), the cantons, inter-communal structures, and the Confederation, are also involved. The final section is a presentation of data collection and sorting methods.

### 2.1 Selection of regional area

For a vast majority of Switzerland's population, three main types of areas constitute the living environment, and generate differing, representative land use policy issues [9]:

• Rural peri-urban regions, which face broad low-density building zone extensions, and where the communes maintain wide building zone reserves;
• Urban regions, which face densification challenges, land reconversion, and remediation issues;
• Mountainous tourist regions: these tend to have a high rate of secondary homes, vast building zone reserves, and ecological challenges linked to tourism and sports.

The areas considered include one rural region, Oberaargau, in Canton Bern, and one urban region, Lausanne, in Canton Vaud. Although no cases stem from the mountainous tourist regions, some of the land use policy issues faced in the touristic areas are comparable to those of rural and peri-urban ones: reduction of over-sized building zones, extensive soil use (due to the construction of numerous secondary homes), and nature protection issues linked with tourism (see [10], [11]).

### 2.2 Selection of local area and cases

The narrower areas are defined based on the political-administrative entities in charge of land-policy implementation. In Canton Bern and Canton Vaud, the communes hold this responsibility for land-policy implementation.

A case law review of land use policy decisions in Bern provided an overview of the conflictual land use policy implementation cases. A keyword analysis of national and regional newspaper archives brought to light land use policy issues that attracted media attention in given local areas. In addition, I conducted preliminary interviews and consulted the cantonal spatial planning archives.

Based on the policy problems related to land use changes, and actor strategies observed within the local areas, I identified twelve cases. Cases are defined as land use policy processes in which authorities face, in a specific context, a specific policy problem, and attempt to solve that problem through an intervention on the target group identified as the cause of that problem. The interactions between authorities and target group. i.e. the strategies adopted, and the resources and policy instruments mobilized, result in a location and time specific arrangement that provides a response to the initial problem. Depending on the type of resolution, the arrangement implements, circumvents, diverts, or innovates land use policy objectives. I applied the criteria defined in the research project within which this research was conducted in order to select appropriate cases [9]:

• Significant changes in land use planning and land use over the past 25 years;
• Presence of oversized building zones;
• Different property rights settings (private, public, reshaping of plots);
• Major land use changes;
• Important issues around ecological and economic added/reduced value;
• Presence of conflicts;
• Use of a specific land use policy instrument.

The research protocol for each case consisted of a detailed description of land use policy changes and of the markers of the theoretically induced explanatory factors. The aim of these descriptions was to identify "trajectories of change and causation" [12:823] through a focus "on sequential processes within a particular historical case" [13:13]. The method used for providing explanations is a mix of what P. Hall [14:25] calls multivariate and theory-oriented explanation: the former refers to the identification "of a small set of variables that can be said to cause such outcomes in a general class of times and places"; the latter tests a theory and attaches special importance in order to "identify the most important elements in the causal chain generating this class of outcomes". Topographic, locational, and soil-related aspects were considered as contextual factors.

### 2.3 Data collection and sorting

For each perimeter considered, I conducted document research, consisting of a review of all land use policy documents elaborated by the commune over the past 25–30 years. Among these documents, one could find:

• Preliminary exam documents, or planning documents submitted by the communes to the canton prior to any revision of their building regulations. Among these documents:
  ○ (Preliminary) Reports on past and future communal development;
  ○ New building regulations as proposed by the commune (structure plans,
  ○ Construction prescriptions, zoning plans, landscape and nature protection plans, equipment plans);
  ○ Reports on the new building regulations justifying how the context changes and how the revised regulations are tackling these changes;
  ○ Relevant protocols and presentations of the communal executive body and legislative body modifying and approving the new documents;
  ○ Oppositions raised by landowners, organizations and other persons allowed to and the relevant protocols of following negotiations (if available);
  ○ Reports of the cantonal planning office and other offices involved in the approval procedure;
  ○ Written correspondence between the canton, commune, urban planner and
  ○ Other actors and notes relating to given phone calls;
  ○ Report on public consultation procedure;
• Approval documents such as approval decisions or decrees of the cantonal authority, final building regulations;
• Potential oppositions and appeals against communal and cantonal decisions;
• Grey literature published by communal, cantonal services, or federal offices (spatial planning offices, water protection office, finance department, audit reports, etc.);
• Professional literature, for example, for the evolution of land prices in the analysed perimeters.

Among the interviews conducted, the following representatives were interviewed for each of the five perimeters analyzed:

• the planner contracted by the commune for establishing the local development plans and revising the building regulations;
• the chief of the communal executive body and/or the communal administrator in charge of planning and construction, (general administration of the commune);
• the cantonal planner in charge of approving communal land use planning documents and her/his co-workers (legal officer, planner from the section dedicated to cantonal or regional land use planning);
• other cantonal offices involved (office for water protection, economic promotion, energy, etc.);
• third parties, such as environmental organizations, landscape protection associations, and journalists.
